# Pathology and Pathogenesis of Eurasian Blackbirds (*Turdus merula*) Naturally Infected with Usutu Virus

**DOI:** 10.3390/v13081481

**Published:** 2021-07-28

**Authors:** Giuseppe Giglia, Gianfilippo Agliani, Bas B. Oude Munnink, Reina S. Sikkema, Maria Teresa Mandara, Elvio Lepri, Marja Kik, Jooske Ijzer, Jolianne M. Rijks, Christine Fast, Marion P. G. Koopmans, Monique H. Verheije, Andrea Gröne, Chantal B. E. M. Reusken, Judith M. A. van den Brand

**Affiliations:** 1 Division of Pathology, Department of Biomedical Health Sciences, Faculty of Veterinary Medicine, Utrecht University, 3584 CL Utrecht, The Netherlands; g.giglia@uu.nl (G.G.); g.agliani@uu.nl (G.A.); m.kik@uu.nl (M.K.); J.Ijzer@uu.nl (J.I.); M.H.Verheije@uu.nl (M.H.V.); A.Grone@uu.nl (A.G.); 2 Department of Veterinary Medicine, University of Perugia, 06126 Perugia, Italy; maria.mandara@unipg.it (M.T.M.); elvio.lepri@unipg.it (E.L.); 3 Department of Viroscience, Erasmus Medical Center, 3000 CA Rotterdam, The Netherlands; b.oudemunnink@erasmusmc.nl (B.B.O.M.); r.sikkema@erasmusmc.nl (R.S.S.); m.koopmans@erasmusmc.nl (M.P.G.K.); chantal.reusken@rivm.nl (C.B.E.M.R.); 4 Dutch Wildlife Health Centre, Utrecht University, 3584 CL Utrecht, The Netherlands; J.M.Rijks@uu.nl; 5 Institute of Novel and Emerging Infectious Disease, Friedrich-Loeffler Institut, D-17493 Isle of Riems, Germany; christine.fast@fli.de; 6 Centre for Infectious Disease Control, National Institute for Public Health and the Environment, 3720 BA Bilthoven, The Netherlands

**Keywords:** Usutu virus, pathology, tissue tropism, Eurasian blackbirds, immunohistochemistry, avian malaria, plasmodium

## Abstract

The Usutu virus (USUV) is a mosquito-borne zoonotic flavivirus. Despite its continuous circulation in Europe, knowledge on the pathology, cellular and tissue tropism and pathogenetic potential of different circulating viral lineages is still fragmentary. Here, macroscopic and microscopic evaluations are performed in association with the study of cell and tissue tropism and comparison of lesion severity of two circulating virus lineages (Europe 3; Africa 3) in 160 Eurasian blackbirds (Turdus merula) in the Netherlands. Results confirm hepatosplenomegaly, coagulative necrosis and lymphoplasmacytic inflammation as major patterns of lesions and, for the first time, vasculitis as a novel virus-associated lesion. A USUV and *Plasmodium* spp. co-infection was commonly identified. The virus was associated with lesions by immunohistochemistry and was reported most commonly in endothelial cells and blood circulating and tissue mononucleated cells, suggesting them as a major route of entry and spread. A tropism for mononuclear phagocytes cells was further supported by viral labeling in multinucleated giant cells. The involvement of ganglionic neurons and epithelial cells of the gastrointestinal tract suggests a possible role of oral transmission, while the involvement of feather follicle shafts and bulbs suggests their use as a diagnostic sample for live bird testing. Finally, results suggest similar pathogenicity for the two circulating lineages.

## 1. Introduction

Usutu virus (USUV) is a zoonotic arbovirus (genus Flavivirus, family Flaviviridae) infecting birds and humans [1,2,3,4]. In birds, it is reported as a cause of mass mortality, mainly affecting passerines and birds of prey. Among passerines, Eurasian blackbirds (Turdus merula) are over-represented, while among birds of prey, the great grey owl (Strix nebulosa) is a commonly affected species [1,4,5,6]. In humans, infection is often asymptomatic or clinically mild and reported as a flu-like syndrome. Nevertheless, occasional reports of a neuroinvasive form of the disease with associated neurological symptoms have been described. USUV is mainly transmitted among birds and from birds to humans by mosquitoes during the blood meal [7]. Occasional detection of viremia in asymptomatic donors suggests that transmission through organ transplants and blood transfusions is a public health concern [8,9,10,11,12,13]. In addition, the co-circulation with closely related flaviviruses, such as the West Nile virus (WNV), as well as the presence of cross-reactivity in serological diagnostics for arbovirus infections, increase the concern on the aspects of surveillance and control of these viral infections in humans [14,15]. Although the first description of the USUV infection in Europe as a cause of mortality in birds dates back to two decades ago (Italy, 1996, and Austria, 2001) [16], most outbreaks have been described in recent years in other European countries. In the Netherlands, episodes of USUV related mortality in passerines and raptors were first described in 2016 [1] and genomic analysis has shown that the virus continues to circulate [17]. Pathogenesis and pathology of USUV in birds have been initially investigated in the early USUV studies [4,5,16,17,18,19,20]. Macroscopically, hepatomegaly and splenomegaly have been reported. Microscopically, lesions were described in the liver, the spleen and the heart, as foci of coagulative necrosis and variable degree of lymphocytic or lympho-histiocytic inflammation [1,5,18,21]. Regarding USUV tissue and cell tropism, the same reports highlight the presence of the viral antigen in the major affected organs (liver, spleen, heart, brain) [5,21,22,23]. Despite the identification of different circulating virus lineages [17], studies investigating their pathogenetic potential in naturally-infected birds are lacking and it has only been partially studied in vitro and in experimental in vivo studies in mice [24,25,26,27,28]. Consequently, due to the small number of animals examined in previous studies, knowledge on the pathology and the complete cell and tissue tropism of the virus, as well as the pathogenetic potential of different circulating viral lineages, are still fragmentary. The goal of this study was to assess, in previously examined organs (e.g., liver, spleen, heart) and not yet examined organs (e.g., skin), the macroscopic and histological changes in a large number of naturally infected Eurasian blackbirds in the Netherlands from 2016–2018. In addition, to better determine the tissue and cell tropism, the presence of virus antigen was investigated on immunohistochemistry (IHC) in all organ systems. Furthermore, as previously reported in this species, we here describe the rate of co-infection with Plasmodium spp. [1]. Finally, a scoring system was developed to assess the severity of histological lesions and to unravel a possible different pathogenicity for the circulating viral lineages in naturally infected Eurasian blackbirds.

## 2. Materials and Methods

### 2.1. Animals

Eurasian blackbirds (Turdus merula) (*n* = 160) that were found dead were collected and submitted to the Dutch Wildlife Health Centre (DWHC) in Utrecht, the Netherlands, as part of an active surveillance program for USUV and West Nile viruses in wild birds (2016–2018). The animals underwent complete necropsy, concurrently with a standard protocol of anamnesis, signalment (sex, age category) and body weight data collection (*n* = 146 animals only). On gross post-mortem examination of the organs, the macroscopic findings were registered, and samples were collected for virological testing and histopathology.

### 2.2. Virology

#### Virus Detection and Sequencing

Brain tissue was collected and stored at −80 °C during necropsies until total nucleic acid (NA) extraction. Tissue was homogenized using the Fastprep bead beater (4.0 m/s for 20 s). Phocine distemper virus (PDV) was added as internal NA extraction control and total NA was extracted from the supernatant using the Roche MagNA Pure. The NA was screened for the presence of USUV using real-time RT-PCRs as described by Nicolay et al. [29] and Jöst et al. [30], in duplex with PDV. USUV lineages were determined by sequencing exactly as described previously [31].

### 2.3. Pathology

During the necropsy, samples from different organs (heart, liver, spleen, lung and air sacs, brain, kidney, glandular and muscular stomach, intestine, pancreas, gonads, skin, thyroid, thymus, and bursa of Fabricius) were collected and fixed in 10% neutral buffered formalin. After routine processing and paraffin embedding, 3–5 μm sections were routinely stained with Haematoxylin and Eosin (H&E) for final examination on light microscopy. Histologic examination of all tissues was performed, and lesions were recorded. For the major affected organs (liver, spleen, heart, brain, lung) a scoring system was defined on proposed literature criteria [32] to evaluate necrosis and inflammation as the most commonly histologic patterns of lesions. For the coagulative necrosis and the inflammation reported in liver, heart, spleen, as well as for the necrosis in the cerebrum (acidophilic neuronal necrosis and/or malacia), the following severity grades were defined as the estimated percentage of the tissue affected on the entire organ section: absent (normal tissue; grade 0), mild (<20%; grade 1), moderate (20–60%; grade 2), severe (>60%; grade 3). For the brain, an additional specific scoring system was established for the perivascular cuffing and the vasculitis per slide, as follows: absent (normal tissue; grade 0), mild (<5 vessels affected; grade 1), moderate (6–20 vessels affected, grade 2), severe (>21 vessels affected; grade 3).

### 2.4. Immunohistochemistry (IHC)

For the detection of USUV antigen in tissues, sections of animals with available tissues (*n* = 147) were stained with a rabbit serum (U433, rabbit vaccinated with inactivated USUV lineage Germany) [22]. Briefly, 3–5 μm tissue sections were deparaffinated and antigen retrieval was performed at 97 °C for 20 min in a citrate buffer (pH 6.0). Blockage of endogenous peroxidase was performed and incubation with the polyclonal rabbit serum was carried out at room temperature for 1 h at 1:1500 dilution. The incubation with the secondary antibody (BrightVision Poly-HRP-Anti Ms-Rb IgG) was performed for 30 min. Finally, an AEC and subsequently a haematoxylin counterstain were applied on the slides and a coverlid was mounted with an aqueous mounting medium (Aquatex). During each staining procedure, 3–5 μm slides from formalin-fixed and paraffin-embedded cytoblocks from the USUV-infected (USUV, Turdus Merula Netherlands 2016; European Virus Archive global (EVAg) Vero cell CCL81 and Vero cell E6 were used as positive control while tissue sections from USUV RT-PCR negative blackbirds were used as negative control.

On IHC, a two-parameter scoring system was defined to assess the grade of labeling in the different examined tissues based on the scoring criteria proposed in the literature [32]. The parameters used for the scoring consisted of: (1) the number of positive cells in 10 random high-power fields (field diameter of 0.55 mm, HPF area of 0.237 mm^2^) and (2) the distribution of the signal in the tissue. For the first parameter, scores were allocated as follows: 0 points, when no signal was identified; 1 point, when 1–10 labelled cell(s) were counted; 2 points, when 11–20 labelled cells were counted; 3 points, when >21 labelled cells were counted. For the second parameter, scores were allocated as follows: 1 point for a focal distribution of signal; 2 points, for a scattered distribution of the signal; 3 points, for a multifocal-patchy distribution of the signal; 4 for the diffuse distribution of the IHC signal. Finally, scores of the two parameters (*n*. positive cells and distribution) were summed and cases were graded as follows: grade 0, when 0 was the total score assigned; grade 1 (low), when 2–3 points were assigned in total to the examined tissue; grade 2 (moderate), when 4–5 points were assigned to the examined tissue; grade 3 (high), when 6–7 points were assigned in total to the examined tissue. To further assess the involvement of specific cell type in tissues, a semiquantitative scoring and morphological identification was performed considering each cell type as follows: + (low; <25% cells infected); ++ (medium; 20–50% cells infected); +++ (high; <50% of cells infected) [32,33].

### 2.5. Statistical Analysis

All statistical analyses were performed using SPSS 26.0 software (IBM, SPSS Inc., Chicago, IL, USA) and statistical significance was set at *p* ≤ 0.05. In order to compare the means of body weight between infected and non-infected animals, a Student’s t-test was performed. A Chi-squared test (χ2) was performed to evaluate the association between sex, age category, common macroscopic findings (e.g., hepatomegaly, splenomegaly) and USUV infection. The Phi Coefficient (ϕ) was next calculated to measure the strength of association. In addition, a Mann–Whitney U test was performed to compare differences in the histology scores for the identified viral lineages, and a Spearman’s correlation was performed to assess the correlation between histology score of the lesions and the IHC score for viral antigen in liver, spleen, heart, brain and lung. The strength of correlation was defined as “high” when absolute value of ρ > 0.5, “medium/moderate” when ρ ranged from 0.3 to 0.49, and “low” when ρ < 0.3 [34].

## 3. Results

### 3.1. Animals

One hundred and sixty Eurasian blackbirds (Turdus merula) were submitted to the DWHC. Most of the animals were reported as “found dead” (*n* = 146) or as “laying on the ground” and/or “unable to fly/falling on a side” (*n* = 14). In the animals found alive, neurological signs as “opisthotonos and/or seizures” and “paralysis” were also reported (*n* = 12/14). The animals were distributed in the age categories using morphologic criteria as follows: juvenile (*n* = 48), adult (*n* = 98), unknown (*n* = 14); while sex distribution was as follows: male (*n* = 93), female (*n* = 45), unknown (*n* = 22). The mean of the animals’ body weight was 88.74 ± 16.36 g (*n* = 146) [35].

### 3.2. Virology

#### Virus Detection and Lineage Determination

USUV RT-PCR was performed and USUV RNA was detected in brain tissue of 118 of 160 birds. The infecting USUV lineage could be determined by whole genome sequencing for 104 birds. Lineage Africa 3 was found in 80 birds and lineage Europe 3 in 24 birds (Table 1).

### 3.3. Pathology

#### 3.3.1. Macroscopic and Histologic Findings

**Figure 1:**
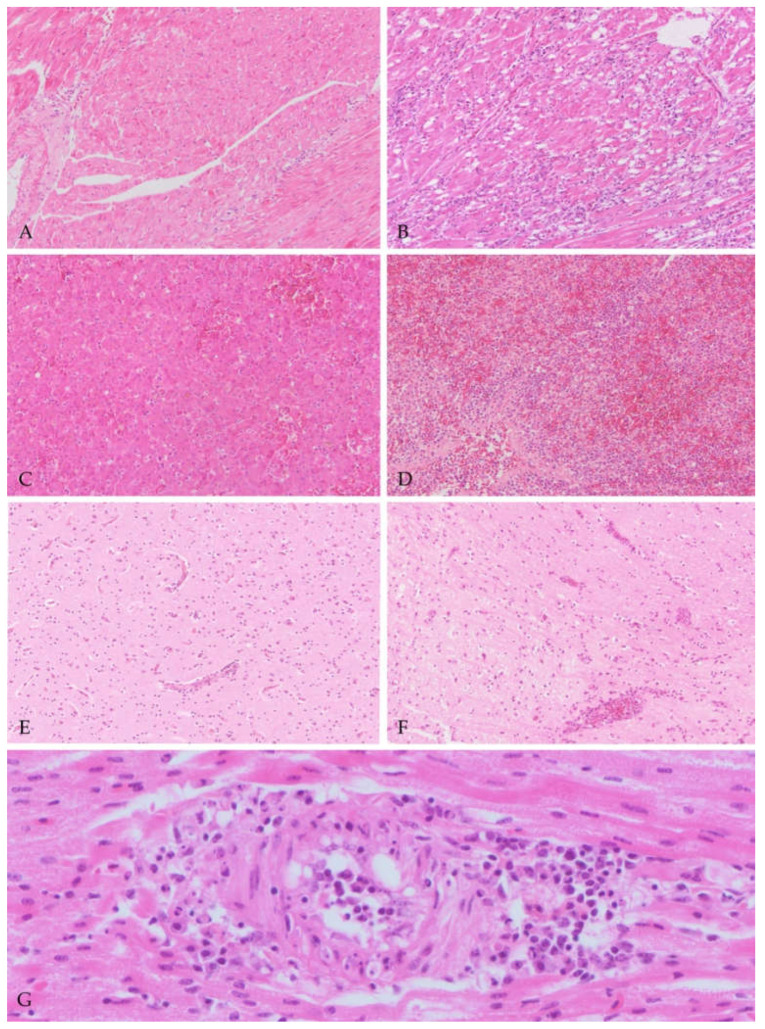
Lesion severity (grades) in different organs and vasculitis in tissues of blackbirds naturally infected with USUV. (**A**,**B**) Heart (20): mild (**A**—Grade 1) and severe (**B**—Grade 3) necrosis of cardiomyocytes and inflammation; (**C**,**D**) liver (20×): mild (**C**—Grade 1) and severe (**D**—Grade 3) necrosis of hepatocytes and inflammation; (**E**,**F**) cerebrum (20×): mild (**E**—Grade 1) and severe (**F**—Grade 3) degree of inflammation represented as perivascular aggregates of mononucleated cells and in the severe case, also rarefaction of the neuropil; (**G**) vasculitis in a cardiac medium caliber artery (40×). Distortion of the vessel wall, with plump and vacuolated endothelial cells, cellular debris and transmural infiltration of inflammatory cells.

At necropsy, on the 160 examined birds, the most frequent gross findings were hepatomegaly (*n* = 125), splenomegaly (*n* = 127), pulmonary hyperemia (*n* = 64), cloacal hyperkeratosis (*n* = 61) and meningeal hyperemia (*n* = 8). On histology, a wide range of findings were identified in different organs; the most common are summarized in Table 2. In the liver, multifocal to coalescing areas of coagulative necrosis (*n* = 118) were commonly identified, often in association with periportal and randomly distributed inflammatory infiltrates. The latter were mainly composed of a variable number of small mature lymphocytes and macrophages (*n* = 118) with occasional plasma cells and heterophils. In the spleen, multifocal areas of necrosis (*n* = 118), commonly in the periarterial sheaths, as well as mononucleate cell (MNC) infiltrates altering the normal tissue architecture were detected (*n* = 85) (splenitis). In the heart, multifocal areas of coagulative necrosis (*n* = 97) and mononucleate cells (MNCs) infiltration (*n* = 126) were recorded. In addition, occasionally in the heart, medium caliber arteries (100 µm in diameter) showed, in the vascular wall, infiltration of lymphocytes in association with endothelial swelling and loss (*n* = 3) (vasculitis). In the kidneys, infiltrates of lymphocytes and plasma cells (*n* = 30) were seen, as well as multifocal foci of acute tubular necrosis (*n* = 16); additional changes such as vasculitis (*n* = 1) and granulomas (*n* = 1) were rarely identified. In the lung, pulmonary edema (in air capillaries and pulmonary interstitium) and a diffuse infiltration of MNCs in the interalveolar interstitium (interstitial pneumonia) (*n* = 128) were seen. In the gastrointestinal tract, the proventriculus showed perineural or serosal perivascular or in the muscular wall randomly distributed infiltrates of MNCs (*n* = 27), while in the gizzard, MNCs were more often recorded in the muscular layer (*n* = 39), occasionally with perineural orientation. A focus of vasculitis was also identified (*n* = 1). In the small and large intestine, MNCs were seen in variable number, expanding and infiltrating the lamina propria (*n* = 37). In the cerebrum, areas of necrosis (*n* = 59) were commonly seen in association with a variable degree of lymphocytes forming perivascular cuffs (*n* = 104), and/or multifocal foci of gliosis. In a large number of cases (*n* = 69), foci of vasculitis were also detected in the cerebrum. In the cerebellum, similar lesions were recorded, mainly involving the molecular layer of cerebellar folia. In the examined cloacal skin, the epidermis showed multifocal areas of mild hyperkeratosis (ortho- and parakeratotic) (*n* = 81) with ulceration and sero-cellular crusts formation (*n* = 42); in the dermis, MNCs were commonly recorded (*n* = 63). Additionally, in few cases, foci of dermal vasculitis (*n* = 2) and feather folliculitis (*n* = 1) were also identified. In the thyroid gland, a parenchymal infiltration of MNCs (*n* = 12) was recorded. Only one case showed a mild hyperplasia of follicular cells (*n* = 1), and a case showed the presence of a granuloma (*n* = 1). In the adrenal gland, microscopic lesions in the parenchyma were rare, with rare MNC infiltrates (*n* = 4). Occasionally, pericapsular MNC infiltrates were visible in the adrenal glands (*n* = 5), often with a perineural orientation (*n* = 4). In the pancreas, autolysis was common and only rare MNC infiltrates were identified (*n* = 2). For the genital system, no microscopic lesions were detected in the testis and ovaries. In the thymus, no microscopic lesions were detected, while in the bursa of Fabricius heterophil and MNCs infiltrations were detected in a single case (*n* = 1). No lesions were detected in the skeletal muscles. Regarding the lesions scores (Figure 1), frequencies and percentages are reported for different grades in Table 3.

In addition to previously mentioned microscopic findings, a high rate of co-infections was also recorded histologically. In particular, in a high number of animals (*n* = 81/160; 50%) extra-erythrocytic stages of hemoprotozoa with morphology suggestive of Plasmodium sp. (Avian Malaria) were seen predominantly in lung, brain and liver. The majority of these cases were also infected with USUV (*n* = 67/119; 56%). Less frequently, mycotic granulomas in the lung (*n* = 8) were seen, few of which being in birds infected with USUV (*n* = 6/119; 5%), as well as numerous circulating microfilariae in blood vessels (*n* = 9) were also identified in USUV infected animals (*n* = 5/119; 4%).

#### 3.3.2. Immunohistochemistry

**Figure 2:**
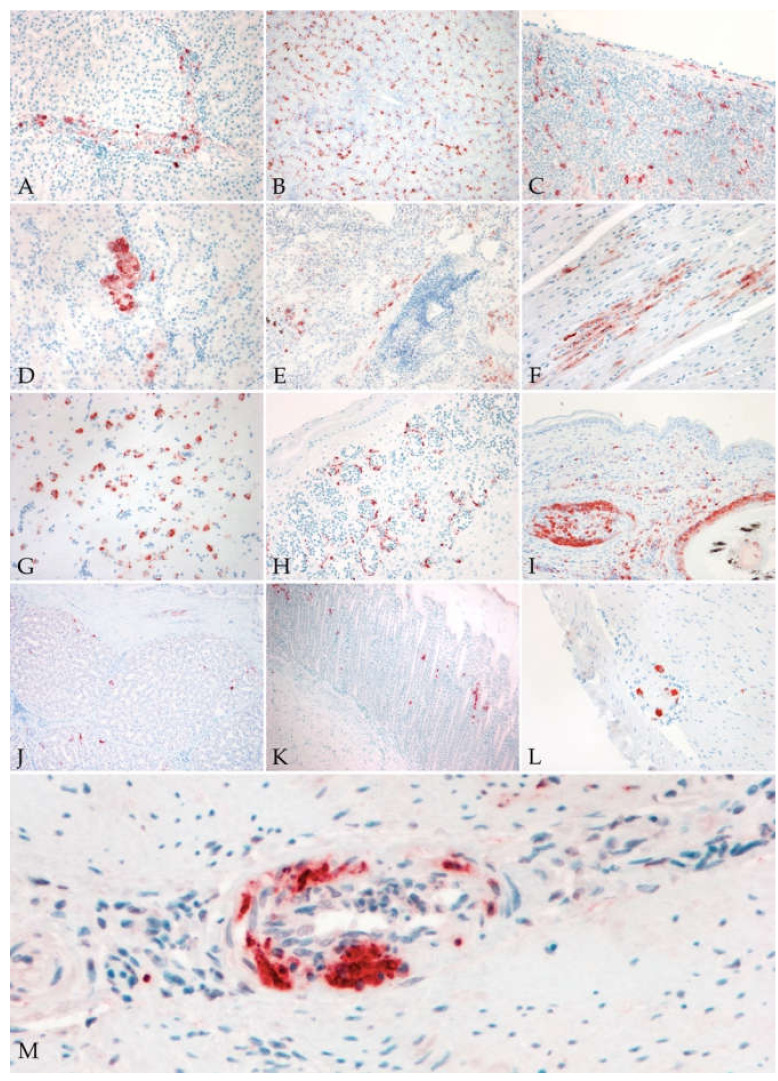
Virus antigen in different tissues of blackbirds naturally infected with USUV. (**A**) Liver: positive intravascular mononucleated cells (20×); (**B**) liver: diffuse presence of virus antigen in Kupffer cells and hepatocytes (10×); (**C**) spleen: diffuse distribution of virus antigen in mononucleated cells and in smooth muscle and fibroblast of the splenic capsule (10×); (**D**) kidney: focal positivity of tubular epithelium (40×); (**E**) lung: multifocal positive pneumocytes, mononucleated inflammatory cells and smooth muscle cells of a pulmonary vessel (10×); (**F**) heart: focal positivity of cardiomyocytes; (**G**) cerebrum: diffuse positivity of neurons and glial cells (40×); (**H**,**I**) skin: diffuse positivity of endothelial cells (**H**; 20×) and positive feather shafts and bulbs (**I**; 20×); (**J**) proventriculus: scattered positive mucosal epithelial cells (10×); (**K**) gizzard: multifocal areas with positive mucosal epithelial cells (10×); (**L**) intestine: positive ganglionic neurons of myenteric plexus (20×); (**M**) medium size artery: positive signal in endothelial cells and in the infiltrating inflammatory cells (vasculitis) (40×).

At immunohistochemistry, USUV antigen was identified in RT-PCR positive birds, in all organs, in endothelial cells and blood circulating (Figure 2A) or tissue MNCs. The antigen was also detected in various organ-specific cells and tissues (Table 4); the number of positive tissues is reported on the total of available examined organs. In the liver (*n* = 78), immunolabeling for the antigen was observed in Kupffer cells and hepatocytes (Figure 2B). In the spleen (*n* = 67), virus antigen was seen in spindle cells of the capsule (pericytes/smooth muscle cells), and in endothelial and smooth muscle cells of the wall of medium caliber arteries and high walled veins (Figure 2C). In the bursa of Fabricius (*n* = 4), only inflammatory MNCs were positive. In the kidney (*n* = 82), viral antigen was observed mainly in the tubular epithelial cells, less commonly in glomeruli (Figure 2D). In the lung (*n* = 59) and air sacs (*n* = 7), type I pneumocytes and spindle cells surrounding the parabronchus or in the air sacs stroma were positive (Figure 2E). When granulomatous lesions were seen in the liver and lungs, multinucleated giant cells (MGCs) were always labeled for the antigen. In the heart (*n* = 88), cardiomyocytes and occasionally endothelial cells of endocardium and epicardium (Figure 2F) were labelled for the virus antigen. In the cerebrum, neurons in the pallium showed intense cytoplasmic staining, as well as glial cells (Figure 2G) and MNCs in the perivascular cuffs. In the cerebellum, the neurons of all three layers of the cerebellar folia as well as the glial cells were positive for the antigen. In the skin (*n* = 55), in association with positive endothelial cells of dermal vessels (Figure 2H), feather follicles showed antigen in the pulp and shafts (Figure 2I), and rare positive keratinocytes in the cloaca. In the thyroid gland (*n* = 21) the epithelial cells lining thyroid follicles were positive. In the gastrointestinal (GI) system the mucosal epithelial cells of proventriculus (*n* = 26) and gizzards (*n* = 38) (Figure 2J,K), and spindle cells of muscular layers resembling both, fibroblasts and smooth muscle cells were positive. Through the intestinal tract (*n* = 39), crypts and villar epithelium, as well as ganglionic neurons in myenteric plexus were positive (Figure 2L). In the pancreas (*n* = 9), epithelial cells of the exocrine component were occasionally positive. In the gonads (*n* = 21), virus antigen was detected in Sertoli’s and germinal cells, while in the ovary, follicular cells in the zona granulosa were positive as well as the occasional spindle cells in the stroma. In a single examined skeletal muscle (*n* = 1), viral antigen was showed only in rare infiltrating MNCs. No adrenal tissue was available for examination in the IHC slides. In all cases of vasculitis, viral antigen was detected in endothelial cells, smooth muscle cells and in infiltrating MNCs (Figure 2M). Virus antigen was not detected in the thymus. Regarding the immunohistochemical grades, frequencies and percentages are reported for each grade in Table 5. Data on severity grades are available in the Supplementary File S1.

#### 3.3.3. Statistical Analysis

Results of the Student’s *t*-test showed a lower body weight in the USUV infected animals (*n* = 105) (M = 84.78 gr; SD = 12.92 gr) compared to the non-infected animals (*n* = 41) (M = 98.91 gr; SD = 19.71 gr) (t(144) = 5.07, *p* < 0.001). The Chi-squared test of independence showed no significant association between age or sex in animals with the USUV infection (*p* > 0.05). The Chi-squared test of independence showed a significant association between the hepatomegaly (*n* = 125) (X(1) = 17.374, *p* < 0.001), the splenomegaly (*n* = 127) (X(1) = 20.825, *p* < 0.001), the cloacal hyperkeratosis (*n* = 61) (X(1) = 18.237, *p* < 0.001), the pulmonary hyperaemia (*n* = 64) (X(1) = 3.965, *p* = 0.046) and USUV infection, while data regarding meningeal hyperaemia (*n* = 8) were not statistically significant. The Phi Coefficient (ϕ) showed that USUV infected animals are more likely to have hepatomegaly (ϕ = >0.338 *p* < 0.001), splenomegaly (ϕ = > 0.370 *p* < 0.001) and cloacal hyperkeratosis (ϕ = >0.346 *p* < 0.001), than the non-infected ones. None of the examined parameters were statistically significant for the Mann–Whitney U test to compare the severity of lesions between the two identified viral lineages (Europe 3 and Africa 3) (*p* > 0.05). The Spearman’s correlation performed to assess the relationship between histology score of the lesions on the available examined tissues and the IHC score of viral antigen expression, showed a moderate statistically significant correlation between the hepatocellular necrosis grade (*n* = 133) and the IHC score for the viral antigen (rs = 0.403, *p* < 0.001). A moderate statistically significant correlation was also observed between both the myocardial necrosis (*n* = 132) (rs = 0.438, *p* < 0.001) and myocarditis (*n* = 132) (rs = 0.398, *p* < 0.001) and the IHC score for the viral antigen. Moreover, the moderate correlation between splenic necrosis and the IHC score for the viral antigen was statistically significant (*n* = 122) (rs = 0.484, *p* < 0.001). For the considered lesion patterns in the central nervous system, the vasculitis (*n* = 135) (rs = 0.531, *p* < 0.001) and the encephalitis (*n* = 135) (rs = 0.480, *p* < 0.001), but not the necrosis (*n* = 135) (rs = 0.217, *p* = 0.012), showed a moderate statistically significant correlation with the IHC score for the viral antigen.

## 4. Discussion

The Usutu virus causes mass mortality in avian species and leads to asymptomatic to mild or neuroinvasive forms of disease in humans [1,3]. In birds naturally infected with USUV, knowledge about pathology and cell and tissue tropism is still fragmentary. The present study describes hepatosplenomegaly, areas of necrosis and mononucleated infiltrates with USUV antigen-positive hepatocytes, endothelial and mononucleated cells as pathologic findings in a USUV infection. Vasculitis is found as a novel virus-associated pattern of lesion in Eurasian blackbirds; insights on multiple transmission routes and the presence of virus antigen in feather shafts and bulbs suggest feathers as a possible diagnostic matrix for testing in live birds.

In accordance with the literature, these results confirm that hepatomegaly and splenomegaly are associated with a USUV infection and should be considered major macroscopic indications of the viral infection [18,19,21,36,37]. Organomegaly was histologically associated to areas of necrosis and mononucleated infiltrates with virus-positive hepatocytes, endothelial and mononucleated cells. Similar histological patterns of lesions were also seen in the heart and the brain, confirming the coagulative necrosis and mononucleated inflammatory infiltrates as the most suggestive and diagnostic lesion patterns identified for a USUV infection [1,19,21], in the brain also associated with scattered foci of gliosis [1,21,36].

Surprisingly, another common and not yet reported pattern of lesions in the cerebrum for this species was vasculitis, consisting in distortion of the vessel wall, in presence of plump endothelial cells, and in transmural infiltration of mononuclear cells. This finding was also rarely identified in the medium caliber arteries of the heart and the kidney, and has been previously reported only in song thrushes and in other bird species infected with the closely related West Nile virus (WNV) [38,39,40,41]. Regarding the vasculitis, on IHC, viral antigen was seen in the endothelial cells as well as in mononucleated inflammatory and media spindle cells, suggesting direct damage to the vessels as an underlying mechanism mediated by the virus. The tissue necrosis observed could be caused either by the vasculitis and/or the direct damage due to the presence of virus. Similarly, also in humans, cases of West Nile virus-associated vasculitis have been reported, suggesting for flaviviruses a similar spectrum of lesions in amplifying and dead-end hosts [42,43].

The clinical aspect of a USUV infection and its clinical progression in naturally infected animals is still poorly described, and only little information is available in literature describing non-specific (e.g., immobility, apathy, ruffled feather) and neurological signs (depression, torticollis, inability to fly) [5,21]. This study suggests for the first time the presence of seizures; however, since these were occasional observations mentioned by laymen who were not trained as veterinarians, these findings should be studied further. The previously described opisthotonos/torticollis defined them as major neurological findings. Based on the pathological findings, seizures are consistent with the telencephalic involvement in which the alteration of the electrical circuit of signal transmission can relate the involvement of areas of motor control in the pallium [44,45,46]. Cerebellar and brainstem involvement are indicated by the observed torticollis and inability to fly [18,21].

Regarding the comparison of the severity of the histologic lesions for the two identified lineages (Europe 3 and Africa 3), results did not show statistically significant differences, suggesting for both lineages a similar pathogenetic effect in naturally infected animals.

For the first time, this study confirms a significant association between hyperkeratosis of the ventral skin cranially to the cloaca, which was previously reported [1] and the presence of the USUV virus antigen. Epidermal hyperplasia, hyperkeratosis (ortho- and parakeratotic), ulceration and crust formation are resembling what is reported in cases of West Nile cutaneous manifestation in humans [47], while dermal mononucleated cell infiltrates (dermatitis) are similar to what is reported for West Nile in Psittaciformes [48]. Regarding the location of the dermatitis development, this could suggest involvement of the highly vascularized brood-patch [49]. However, the exact mechanism of lesion development is not known. The presence of the virus antigen in cutaneous endothelial cells and mononucleated cells suggests these cells as a major way of entry and spread that may play an important role in the pathogenesis. Further research is needed to clarify whether these lesions are multifocally spread and directly caused by the virus or focal, limited to the ventral cloaca and related to the immobilization of the animals on the ground with underlying mechanical stimulus as cause. The virus antigen was also detected in skin, for the first time, in epithelial cells of the feather shafts and feather bulb, suggesting the possible use of feathers for live-animal testing, as already reported for WNV [50,51], for USUV [52] and highly pathogenic avian influenza [53].

A strong presence of the virus antigen in cells of the mononuclear phagocyte system was highlighted by immunolabeling of Kupffer cells and mononucleated inflammatory cells in various tissues or circulating in blood vessels lumina, as previously reported [18], as well as in multinucleated giant cells in granulomas, described in this study for the first time. Unfortunately, it is not possible to differentiate active infection from phagocytized infected material. For this reasons, these results suggest two possible scenarios: first, viral phagocytosis as part of the normal mechanism of agent clearance in innate immunity from antigen presenting cells; second and more interestingly, a real cell tropism due to cell-mediated entry and active viral replication, as seen for the West Nile virus [54,55,56,57]. These data supports a possible cell-mediated mechanism of viremia, with a so called “trojan horse” mechanism of spread [57,58], but further research is needed to clarify the exact role of monocytes–macrophages in the pathogenesis of this disease, as performed for WNV [56,59].

In the gastrointestinal (GI) tract, lesions were subtle and only presented as mild infiltrates of mononucleated cells in the lamina propria and muscular layers of proventriculus, gizzard and intestine, and the virus antigen was seen in epithelial cells and spindle cells and, as in another single report in blackbirds [60], in neurons of the peripheral myenteric autonomic ganglia. The positivity of GI epithelial cells suggests a possible role in the viral shedding (sloughing off cells from the mucosa) in the environment and a possible alternative route of a direct infection, but both still remains to be clarified. The positivity of ganglionic neurons is suggestive of a virus tropism for the peripheral nervous system, as was suspected in a recent case of USUV-related human peripheral neuropathy [61]. Furthermore, it suggests the possibility of oral infection, due to ingestion of infected mosquitoes (passerines) or infected birds (raptors), and subsequent peripheral entry and spread as suggested for avian influenza in cats and for birds and mammals experimentally infected with WNVs [62,63,64,65].

Regarding additional transmission routes, in this study virus antigen was also reported in the genital system for both testis and ovaries. This finding has been reported for USUV in raptors [5] and for WNV in passerine species [66], and could suggest a possible vertical transmission route of the virus or be an indication of a possible viral-mediated reduction in the individual fertility. For these reasons further investigation is required.

In addition, the extensive evaluation of all organs showed in the thyroid the presence of the virus antigen in follicular cells in the absence of histological changes. To our knowledge, reports of the viral antigen in this organ have not been reported in birds infected by USUV or WNV, while rare reports of thyroiditis are described for West Nile cases in humans [67]. However, the exact effect of flaviviruses on this organ are still to be unraveled. In the present study, the pancreas rarely (*n* = 2) showed mild infiltrates of mononucleated cells with viral antigen seen in the exocrine epithelial component as previously reported [19,68]. In thymus and bursa of Fabricius and skeletal muscles lesions were absent or rare, as well as the presence of the viral antigen. The data suggests the last-mentioned organs as less commonly affected during USUV infection.

Regarding the presence of the virus antigen and its relationship with tissue lesions, results showed that a higher number of positive cells are seen in birds with higher tissue necrosis in liver, heart and spleen, and inflammation in heart, spleen, and cerebrum. This result could potentially suggest a possible indication of the stage and progression of the disease. For this reason, the virus antigen score in IHC could potentially be used as an indication of the disease stage in dead and live birds, but further experiments are needed to clarify changes in lesions severity and viral presence during the disease progression.

In addition to the mentioned findings, a high rate of co-infections was also histologically recorded. A high number of animals showed the presence of extra-erythrocytic stages of hemoprotozoa with morphology suggestive of Plasmodium sp. (avian malaria) concurrent with a USUV infection. This finding has previously been reported in the Netherlands and in Belgium [1,69], but the effect of this co-infection in the disease severity is still unknown and currently under investigation. Less common presence of concurrent mycotic pneumonia and circulating microfilariae suggest a possible impairment of the general health condition of these animals with the development of concurrent secondary mycotic infections, as well as a possible co-transmission of the virus, filariids worms and hemoprotozoa from the same mosquitoes’ species.

Regarding the animals, the results showed that of the 160 found dead Eurasian blackbirds (Turdus merula) examined in this study, the majority were adult (*n* = 98/160), male (*n* = 93/160) individuals. The difference in weight was statistically significant, with infected animals having a lower weight compared to non-infected ones, suggesting a possible anorexic behavior followed by weight loss, commonly associated to conditions of systemic illness and avian viral infections, such as West Nile [52,70,71]. The data regarding association between age or sex categories and viral infection were not statically significant. One explanation to the higher prevalence of these categories of animals in this study can be justified by the easier identification of dead black, adult males on fields by the collectors in comparison with the more mimetic brownish juvenile and female individuals [72].

From a human perspective, this study highlights pathologic features and possible pathogenetic steps in avian species highly susceptible to USUV, suggesting different transmission routes, as well as various common and uncommon patterns of tissue response. Since pathologic descriptions of human cases are lacking, the here described findings in the amplifying host may give insights in the pathogenesis for the human disease, which can be used for the development of future preventive and therapeutical measures.

Finally, this study put additional focus on the importance and necessity of the dead bird’s surveillance to control and prevent outbreaks of flavivirus-associated disease in animals and human.

## 5. Conclusions

In conclusion, the colocalization of lesions and the presence of virus antigen in blood vessels, cells of the mononuclear–phagocyte system and feathers in blackbirds naturally infected with USUV suggests an important role in the pathogenesis of USUV related disease. This study confirms the coagulative necrosis and mononuclear inflammation as major USUV-associated microscopic patterns and highlights the vasculitis as novel virus-associated lesion in Eurasian blackbirds. Furthermore, involvement of endothelial cells as well as circulating and tissue mononucleate cells suggests an important role for these cells in the pathogenesis of USUV infection. The finding of the virus antigen presence in feathers provides evidence for the use of feathers as a possible diagnostic tool for live bird testing.

## Figures and Tables

**Table 1:** Summary of virology data of the Eurasian blackbirds.

	USUV RT-PCR	Ct Values
USUV Positive *	118/160	21.62 ± 4.63
Europe 3	24/118	20.84 ± 4.14
Africa 3	80/118	20.99 ± 3.65

* Not all USUV positive samples were sequenced.

**Table 2:** Major microscopic findings of liver, spleen, hearth, lung and cerebrum in all the available examined tissues of Eurasian blackbirds, the Netherlands, 2016–2018.

Organ	Lesion	N°	%
Liver	Necrosis	118/143	82
	Hepatitis	118/143	82
Spleen	Necrosis	118/142	83
	Splenitis	85/142	59
Heart	Necrosis	97/145	66
	Myocarditis	126/145	86
	Vasculitis	3/145	2
Lung	Pneumonia	128/139	92
Cerebrum	Necrosis	59/145	40
	Perivascular cuffs	104/145	71
	Vasculitis	69/145	47

**Table 3:** Distribution of H&E lesion grades in various most affected organs of blackbirds infected with USUV.

Organ		Grade 0 *		Grade 1		Grade 2		Grade 3	
		N°	%	N°	%	N°	%	N°	%
Liver	Necrosis	25/143	17	77/143	53	35/143	24	6/143	4
	Hepatitis	25/143	17	69/143	48	46/143	32	3/143	2
Spleen	Necrosis	24/142	16	35/142	24	61/142	43	22/142	15
	Splenitis	57/142	40	41/142	28	40/142	28	4/142	2
Heart	Necrosis	48/145	33	62/145	42	32/145	22	3/145	2
	Myocarditis	19/145	13	58/145	40	60/145	41	8/145	5
Lung	Pneumonia	11/139	7	69/139	49	51/139	36	8/139	5
Brain	Encephalitis	41/145	28	52/145	35	27/145	18	3/145	17
	Vasculitis	76/145	52	37/145	25	26/145	17	6/145	4
	Necrosis	86/145	53	48/145	33	10/145	6	1/145	1

Grade 0 = absence of lesion; Grade 1 = mild lesions; Grade 2 = moderate lesions; Grade 3 = severe lesions.

**Table 4:** Overview of the different involvement of the USUV-antigen in cell types in different organs.

Organ	Tissue/Cell	Immunolabeling *
All organs	Endothelial cells Vascular wall Mononucleated cells Intravascular leukocytes	+++ ++ ++ +
Liver	Kupffer’s cells Hepatocytes	+++ +
Spleen	Capsular stromal cells High walled veins	++ +
Heart	Cardiomyocytes Endocardium	+++ ++
Lung	Type I pneumocytes	+
Air sacs	Spindle stromal cells	+
Kidney	Tubular epithelium Glomeruli	+++ +
Skin	Keratinocytes Feathers	+ ++
Cerebrum	Neurons Glial cells	+++ +
Cerebellum	Neurons Glial cells	+++ +
Proventriculus	Mucosal epithelium Smooth muscle cells Peripheral ganglion	++ + +
Gizzard	Mucosal epithelium Smooth muscle cells Peripheral ganglion	++ + +
Intestine	Mucosal epithelium Smooth muscle cells Peripheral ganglion	++ + +
Gonads	Sertoli cells Ovary follicular epithelium Germinal cells	+ + +
Thyroid	Follicular epithelium	++
Skeletal muscle	Stromal cells	+

* + few cells infected; ++ moderate numbers of cells infected; +++ numerous cells infected.

**Table 5:** Distribution of IHC grades in the available tissues of the most commonly affected organs (all percentages rounded off to the bottom) of blackbirds infected with USUV.

Organ	Grade 0		Grade 1		Grade 2		Grade 3	
	N°	%	N°	%	N°	%	N°	%
Liver	59/137	43	35/137	25	10/137	7	33/137	24
Spleen	58/125	46	26/125	20	12/125	9	29/125	23
Heart	45/133	33	25/133	18	10/133	7	53/133	35
Kidney	57/138	41	30/138	21	15/138	10	36/138	26
Lung	69/128	53	25/128	19	9/128	7	25/128	19
Air sacs	7/14	50	3/14	21	0/14	0	4/14	28
Cerebrum	60/136	44	17/136	12	8/136	5	51/136	37
Cerebellum	60/98	61	14/98	14	12/98	12	12/98	12
Skin	27/82	32	6/82	7	4/82	4	45/82	54
Gizzard	88/126	69	10/126	7	9/126	7	19/126	15
Proventriculus	79/105	75	8/105	7	7/105	6	11/105	10
Intestine	65/104	62	4/104	3	11/104	10	24/104	23
Pancreas	22/31	70	6/31	19	0/31	0	3/31	9
Gonads	44/65	67	5/65	7	4/65	6	12/65	18
Thyroids	47/68	69	5/68	7	4/68	5	12/68	17
Bursa of Fabricius	11/15	73	1/15	6	0/15	0	3/15	20
Thymus	0/2	0	0/2	0	0/2	0	0/2	0
Skeletal muscle	0/1	0	0/1	0	1/1	100	0/1	0

## Data Availability

The data presented in this study are available in the Supplementary Material: S1.

## Supplementary Materials

The following are available online at https://www.mdpi.com/article/10.3390/v13081481/s1, File S1: suppl1.
